# Effectiveness of two distinct web-based education tools for bedside nurses on medication administration practice for venous thromboembolism prevention: A randomized clinical trial

**DOI:** 10.1371/journal.pone.0181664

**Published:** 2017-08-16

**Authors:** Brandyn D. Lau, Dauryne L. Shaffer, Deborah B. Hobson, Gayane Yenokyan, Jiangxia Wang, Elizabeth A. Sugar, Joseph K. Canner, David Bongiovanni, Peggy S. Kraus, Victor O. Popoola, Hasan M. Shihab, Norma E. Farrow, Jonathan K. Aboagye, Peter J. Pronovost, Michael B. Streiff, Elliott R. Haut

**Affiliations:** 1 Division of Acute Care Surgery, Department of Surgery, The Johns Hopkins University School of Medicine, Baltimore, Maryland, United States of America; 2 Division of Health Sciences Informatics, The Johns Hopkins University School of Medicine, Baltimore, Maryland, United States of America; 3 The Armstrong Institute for Patient Safety and Quality, Johns Hopkins Medicine, Baltimore, Maryland, United States of America; 4 Department of Health Policy and Management, The Johns Hopkins Bloomberg School of Public Health, Baltimore, Maryland, United States of America; 5 Department of Nursing, The Johns Hopkins Hospital, Baltimore, Maryland, United States of America; 6 Department of Biostatistics, The Johns Hopkins Bloomberg School of Public Health, Baltimore, Maryland, United States of America; 7 Johns Hopkins Surgery Center for Outcomes Research, The Johns Hopkins University School of Medicine, Baltimore, Maryland, United States of America; 8 Department of Pharmacy, The Johns Hopkins Hospital, Baltimore, Maryland, United States of America; 9 Department of Surgery, Duke University, Durham, North Carolina, United States of America; 10 Department of Anesthesiology and Critical Care Medicine, The Johns Hopkins University School of Medicine, Baltimore, Maryland, United States of America; 11 Division of Hematology, Department of Medicine, The Johns Hopkins University School of Medicine, Baltimore, Maryland, United States of America; 12 Department of Emergency Medicine, The Johns Hopkins University School of Medicine, Baltimore, Maryland, United States of America; Universite de Bretagne Occidentale, FRANCE

## Abstract

**Background:**

Venous thromboembolism (VTE) is a common cause of preventable harm in hospitalized patients. While numerous successful interventions have been implemented to improve prescription of VTE prophylaxis, a substantial proportion of doses of prescribed preventive medications are not administered to hospitalized patients. The purpose of this trial was to evaluate the effectiveness of nurse education on medication administration practice.

**Methods:**

This was a double-blinded, cluster randomized trial in 21 medical or surgical floors of 933 nurses at The Johns Hopkins Hospital, an academic medical center, from April 1, 2014 –March 31, 2015. Nurses were cluster-randomized by hospital floor to receive either a linear static education (Static) module with voiceover or an interactive learner-centric dynamic scenario-based education (Dynamic) module. The primary and secondary outcomes were non-administration of prescribed VTE prophylaxis medication and nurse-reported satisfaction with education modules, respectively.

**Results:**

Overall, non-administration improved significantly following education (12.4% vs. 11.1%, conditional OR: 0.87, 95% CI: 0.80–0.95, p = 0.002) achieving our primary objective. The reduction in non-administration was greater for those randomized to the Dynamic arm (10.8% vs. 9.2%, conditional OR: 0.83, 95% CI: 0.72–0.95) versus the Static arm (14.5% vs. 13.5%, conditional OR: 0.92, 95% CI: 0.81–1.03), although the difference between arms was not statistically significant (p = 0.26). Satisfaction scores were significantly higher (p<0.05) for all survey items for nurses in the Dynamic arm.

**Conclusions:**

Education for nurses significantly improves medication administration practice. Dynamic learner-centered education is more effective at engaging nurses. These findings suggest that education should be tailored to the learner.

**Trial registration:**

ClinicalTrials.gov NCT02301793

## Introduction

Venous thromboembolism (VTE), comprised of deep vein thrombosis (DVT) and/or pulmonary embolism (PE), affects 350,000–600,000 individuals in the United States annually. More than 100,000 people die each year in the United States as a result of PE–more than from breast cancer, AIDS, and motor vehicle collisions combined.[1] Despite the availability of effective prophylaxis against VTE,[2] numerous studies have shown that VTE prophylaxis is vastly underutilized in hospitals[3,4] and the Agency for Healthcare Research and Quality (AHRQ) has listed strategies to improve VTE prevention on its top ten list for patient safety practices.[5–8] Consequently, numerous interventions have been implemented to improve prescription of VTE prophylaxis[8–11] with the implicit assumption that medications prescribed for hospitalized patients will always be administered. Few studies have focused on nurses, patients, and the administration of prescribed VTE prophylaxis.[12,13]

Studies from multiple hospitals have shown that 10–12% of prescribed doses of pharmacologic VTE prophylaxis are not administered to hospitalized patients and that the most frequently documented reason for non-administration is patient refusal.[14,15] While patients have the right to refuse any type of care, it is the responsibility of healthcare providers to educate patients so that they can make informed decisions. However, some nurses unilaterally make clinical decisions regarding the appropriateness of prescribed pharmacological VTE prophylaxis and/or allow patients to make uninformed decisions to refuse prophylaxis without educating patients about the harms of VTE or benefits of VTE prophylaxis.[16]

Recent evidence has suggested that missing doses of prescribed pharmacologic VTE prophylaxis is associated with the development of VTE in hospitalized patients.[17,18] As part of a multifaceted approach to directly address missed doses of prophylaxis and decrease preventable harm from VTE, we targeted nurses with education. We conducted a cluster randomized clinical trial of two web-based modules to educate nurses about the harms of VTE, benefits of VTE prophylaxis, and strategies to better communicate this information to patients. Our primary aim was to determine the effectiveness of nurse education to reduce non-administration of prescribed doses of pharmacologic VTE prophylaxis in hospitalized medical and surgical patients. Our secondary aims were to assess the differential effect of different educational approaches and nurse perceptions about educational strategies and dose refusal as well as other reasons for non-administration of prescribed VTE prophylaxis medication.

## Materials and methods

### Trial design

This double-blinded, cluster randomized clinical trial was conducted at the Johns Hopkins Hospital, an academic medical center in Baltimore, Maryland from April 1, 2014 –March 31, 2015. We included 21 adult inpatient floors comprised of internal medicine floors (n = 11) and surgery floors (n = 10). The Johns Hopkins Medicine Institutional Review Board approved the study (IRB00043860), a waiver of consent was provided, and the trial was registered on ClinicalTrials.gov (“Educating Nurses About Venous Thromboembolism (VTE) Prevention” NCT02301793).

### Educational interventions

In partnership with the Johns Hopkins Central Nursing Education, we built two Web-based educational modules about VTE prevention. One arm provided linear static education (Static) using PowerPoint slides with voiceover to cover the concepts. The other arm provided interactive learner-centric dynamic scenario-based education (Dynamic), where each scenario resulted in either positive reinforcement or corrective feedback with an opportunity to apply knowledge to a new scenario. Both education modules were computer-based containing the same general concepts about VTE prevention practices, including harms associated with VTE, incidence of VTE, and best-practices regarding communicating the importance of VTE prophylaxis and how to administer VTE prophylaxis. Additionally, each module took approximately the same amount of time to complete, though no time limit was imposed after initiativing the education module.

### Sample size

Based on an intervention to educate every hospitalized patient about the harms of VTE and benefits of VTE prophylaxis, Piazza et al found a reduction in nonadministration from 10.1% to 5.6% or a 45% reduction in nonadministration.[12] Assuming 45 to 50% relative reduction in non-administration in the intervention group, the effect size in form of the odds ratio is between 0.43 and 0.47. We will have at least 80% power to detect a 60% reduction in odds of missed dose at 0.05 level of statistical significance.

### Enrollment and randomization

Nurses were identified using our centralized education directory that associates nurses with their designated departments and hospital floors. Beginning on July 23, 2014, nurses were cluster randomized by floor to receive one of two education modules about VTE prevention. Nurses were asked to complete their assigned education module by October 23, 2014. All nurses associated with one of the 21 adult inpatient floors were eligible for enrollment in the trial. Nurses who were not permanently associated with one of the 21 hospital floors (e.g. traveling nurse, float nurse) were excluded from this study. Because of known differences between medical and surgical floors in VTE prophylaxis administration practice and culture,[14,16,19–21] floors were stratified by department (i.e. medicine and surgery) for randomization. Within strata, a coin toss (ERH) was used to randomize floors into either the Dynamic education arm or the Static education arm. Based on the outcome of the coin toss, nurses were then remotely assigned the online education module by an institutional nurse educator (DLS). The assigned educational module then appeared in the nurse’s list of education assignments for completion within the institutional Learning Management System. Nurses are required to complete clinically relevant education regularly as part of ongoing professional practice and a waiver of consent was provided by the IRB; therefore nurses were not aware of their participation in a trial nor were they aware that two education modules existed. Additionally, the VTE prophylaxis medication non-administration dataset provided to the biostatistical team (i.e. outcomes assessors) for analysis was blinded by treatment arm and department.

### Data collection

Patient demographic data were extracted from the Johns Hopkins Hospital administrative database. Pharmacologic VTE prophylaxis medication administration data were extracted directly from electronic medication administration record in our computerized provider order entry system. Data were collected for one year and divided into three distinct time periods: April 1 –July 22, 2014 (Baseline); July 23, 2014 –October 23, 2014 (Education Intervention); October 24, 2014 –March 31, 2015 (Post-Education). Immediately following completion of the assigned education module, nurses were asked to complete a voluntary 5-question survey to assess the relevance of and satisfaction with the education module.

### Statistical analysis

Our primary outcome was the proportion of prescribed pharmacologic VTE prophylaxis doses not administered. Secondary outcomes included nurses satisfaction and the reason for non-administration (i.e. patient refusal vs other). The hypothesis comparing the different educational arms was evaluated using an intention-to-treat approach (i.e. all nurses were included regardless of training completion) accounting for clustering in the data and comparing rates of VTE prophylaxis dose non-administration at baseline and during the Post-Education period. Two per-protocol sensitivity analyses were performed. The first compared the Baseline vs. Post-Education periods for those nurses who had received training. The second, which was also limited to those nurses who completed training, allocated visits (including those during the training period) to the Pre- vs. Post-Education periods based upon the training date for each individual nurse.

Our biostatistician team (GY, JW, EAS) were blinded to the intervention arm assignment and department strata. Patient visit-level demographic characteristics for the Baseline period were described by arm. The changes in pharmacologic VTE prophylaxis administration practices between the Baseline and Post-Intervention periods were compared overall and between the two education arms using generalized linear mixed-effects models with a logistic regression model and random intercepts for floor and nurse to account for the intragroup correlation within the same floor or within the same nurse. Due to the complexity of the multilevel structure of the data (i.e. multiple doses per patient across various hospitalizations, nurses and floors), multiple outputation[22] was employed to reduce the levels of hierarchical structure to the floor level and nurse level by randomly selecting one dosage per patient. By reiterating the procedure 1000 times, we estimated the odds ratios (ORs) and their 95% confidence intervals conditional on the floor and nurse. The reported p-values are also derived from this procedure. A test of interaction was used to interpret the comparison of reported outcomes between the two intervention arms. Responses to the follow-up survey to assess nurse perception with the education modules were analyzed using a two-sided Chi-squared test. All statistical tests were conducted at 5% statistical significance. Statistical analyses were performed using Stata version 14.1 MP (College Station, Texas 77845). The Stata.do file (S3 File) and the.log files (S4 File) are included as Supporting Information.

## Results

**Fig 1:**
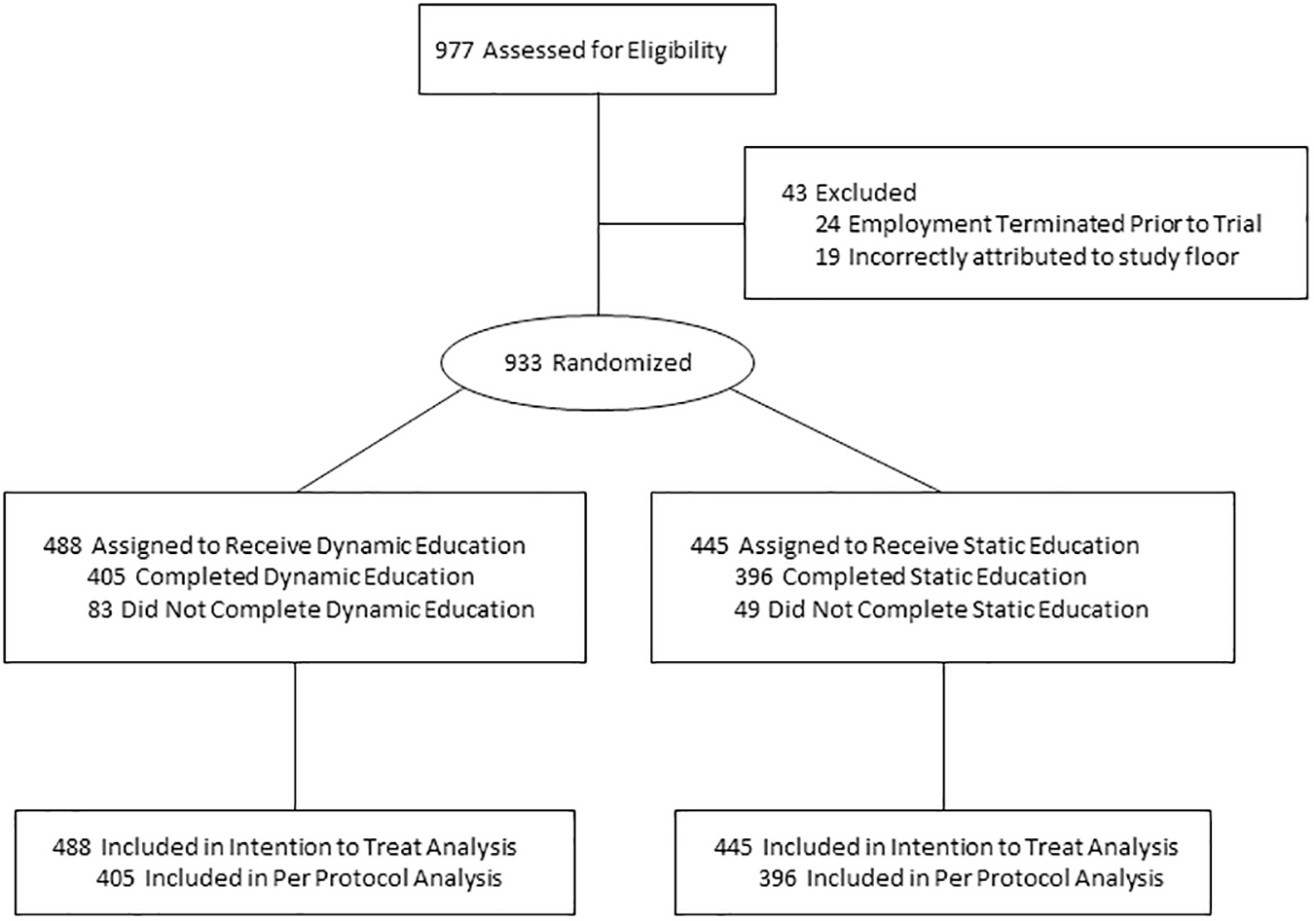
Flow of nurse participants through trial comparing Dynamic education with static education on medication administration practice for venous thromboembolism prevention.

Among the 21 hospital floors included in this study, 11 floors (6 medicine and 5 surgery) were randomized to the Dynamic arm and 10 floors (5 medicine and 5 surgery) were randomized to the Static arm. Out of 977 nurses identified as being potentially eligible for inclusion using our centralized education directory, 933 (95.5%) were determined to be actively employed by The Johns Hopkins Hospital and associated with the study floors (Fig 1). By the end of the education trial period, 396/445 (89.0%) nurses who were assigned had completed the Static module and 405/488 (83.0%) nurses who were assigned had completed the Dynamic module. During the entire study period, 214,478 doses of pharmacologic VTE prophylaxis were prescribed to patients on the 21 hospital floors. During the Baseline period, there were 2,722 patient visits in the Dynamic arm and 2,603 patient visits in the Static arm. The baseline patient demographics are listed in Table 1. Before the trial began, non-administration ranged by floor from 3.2% to 32.7%. After the trial was completed, non-administration by floor ranged from from 3.7% to 35.2%.

**Table 1:** Demographic characteristics of patient visits during the Baseline period, by arm.

	Dynamic Arm (n = 2,722)	Static Arm (n = 2,603)
**Unique Patients**	1,925	2,021
**Mean Age (SD), years**	55.6 (16.9)	56.3 (17.3)
**Age Range, years**	17–102	15–97
**Sex, n (%)**		
Male	1,435 (52.7%)	1,186 (45.6%)
Female	1,287 (47.3%)	1,417 (54.4%)
**Race, n (%)**		
Black	1,106 (40.6%)	980 (37.7%)
White	1,367 (50.2%)	1,396 (53.6%)
Asian	46 (1.7%)	50 (1.9%)
Native American	4 (0.2%)	7 (0.3%)
Other	199 (7.3%)	170 (6.5%)
**Median Number of Prescribed Doses of VTE Prophylaxis Medication per Patient (Q1, Q3)**	7 (3,13)	7 (3,13)
**Median Length of Hospital Stay, days (Q1, Q3)**	4 (2, 8)	5 (2, 8)

### VTE prophylaxis non-administration analyses

Overall, non-administration of pharmacologic VTE prophylaxis improved significantly following the education in the trial (12.4% vs. 11.1%, conditional odds ratio [cOR]: 0.87, 95% CI: 0.80–0.95, p = 0.002, Table 2) achieving our primary objective. The magnitude of the reduction in non-administration was slightly greater in the the Dynamic arm following education (10.8% vs. 9.2%, cOR: 0.83, 95% CI: 0.72–0.95) compared with the Static arm (14.5% vs. 13.5%, cOR: 0.92, 95% CI: 0.81–1.03), although the difference between intervention arms was not statistically significant (p = 0.26). These findings were similar when limiting the analyses to nurses who completed the education and comparing both before and after the trial period and before and after the date when individual nurses completed their assigned training (Table 2).

**Table 2:** Comparison of the pattern of non-administration of prescribed venous thromboembolism prophylaxis medication doses for the dynamic and static education interventions. The pre- and post-education periods are defined either based upon the overall training period (i.e. excluding all visits within the training period regardless of the individual nurses’ training with a common pre- and post- period) or based upon the individual nurses’ training (i.e. includes all visits within the pre- and post- period for each individual nurse).

	Pre-Education % (95% CI)	Post-Education % (95% CI)	Odds Ratio: Post/Pre (95% CI)	Ratio of Odds Ratios: Static/Dynamic (95% CI)	P-value
Intention to Treat*: *Period based upon overall training*					
**Overall**	12.4% (9.6%, 15.9%)	11.1% (8.6%, 14.2%)	0.87 (0.80, 0.95)		0.002§
**Dynamic Education**	10.8% (7.7%, 15.0%)	9.2% (6.6%, 12.8%)	0.83 (0.72, 0.95)		
**Static Education**	14.5% (10.2%, 20.4%)	13.5% (9.6%, 19.1%)	0.92 (0.81, 1.03)	1.11 (0.92, 1.33)	0.26‡
*Per Protocol*†: *Period based upon overall training*					
**Overall**	12.3% (9.6%, 15.7%)	11.2% (8.8%, 14.3%)	0.89 (0.81, 0.95)		0.012§
**Dynamic Education**	10.6% (7.7%, 14.7%)	9.4% (6.8%, 13.0%)	0.86 (0.75, 0.99)		
**Static Education**	14.4% (10.3%, 20.2%)	13.5% (9.7%, 19.0%)	0.92 (0.82, 1.04)	1.07 (0.89, 1.30)	0.438‡
*Per Protocol*†: *Period based upon individual nurses’ training*					
**Overall**	12.1% (9.5%, 15.5%)	10.9% (8.5%, 13.9%)	0.88 (0.81, 0.95)		0.001§
**Dynamic Education**	10.6% (7.6%, 14.7%)	9.3% (6.7%, 12.9%)	0.86 (0.76, 0.96)		
**Static Education**	14.1% (10.0%, 19.8%)	13.0% (9.2%, 18.3%)	0.90 (0.81, 1.01)	1.06 (0.90, 1.24)	0.516‡

*The intention to treat cohort includes all visits with all nurses regardless of whether or not training was completed.

†The per protocol analysis only includes those visits overseen by nurses who received training.

§The p-value compares whether the overall change in the odds of non-administration differs between pre-education and post-education, regardless of arm assignment.

‡The p-value compares whether the change in the odds of missing an administration differs by arm (i.e. a test of interaction between period and arm).

% = percent; CI = confidence interval.

There was no change in the proportion of prescribed doses that were documented as refused by patients or family members overall (cOR: 0.91, 95% CI: 0.81, 1.02, p = 0.113, Table 3) in either the Dynamic arm (5.6% vs. 5.1%, cOR: 0.89, 95% CI: 0.75–1.04) or the Static arm (7.3% vs. 7.0%, cOR: 0.94, 95% CI: 0.81–1.10). Overall, non-administration for other reasons (i.e. patient off the floor, dose held for planned invasive procedure, etc.) was significantly lower after the education trial (4.1% vs. 3.4%, cOR: 0.82, 95% CI: 0.73–0.92, p<0.001). The magnitude of the reduction in non-administration for other reasons was slightly greater in the Dynamic arm (3.4% vs. 2.6%, cOR: 0.73, 95% CI: 0.60–0.88) compared with in the Static arm (5.0% vs. 4.4%, cOR: 0.89, 95% CI: 0.76–1.04, Table 3), although there was no statistically significant difference between intervention arms (p = 0.151).

**Table 3:** Comparison of the reason for non-administration of prescribed venous thromboembolism prophylaxis medication doses for the dynamic and static education interventions. The Pre- and Post-Education periods are defined based upon the overall training period (i.e. excluding all visits within the training period regardless of the individual nurses’ training with a common pre- and post- period).

	Pre-Education % (95% CI)	Post-Education % (95% CI)	Odds Ratio: Post/Pre (95% CI)	Ratio of Odds Ratios: Static/Dynamic (95% CI)	P-value
*Reason for Non-administration*: *Patient or Family Member Refusal*					
**Overall**	6.4% (4.2%, 9.7%)	5.9% (3.9%, 9.0%)	0.91 (0.81, 1.02)		0.113§
**Dynamic Education**	5.6% (3.1%, 10.0%)	5.1% (2.8%, 9.0%)	0.89 (0.75, 1.04)		
**Static Education**	7.3% (4.0%, 13.2%)	7.0% (3.8%, 12.6%)	0.94 (0.81, 1.10)	1.06 (0.85, 1.33)	0.584‡
*Reason for Non-administration*: *Other*					
**Overall**	4.1% (3.3%, 5.0%)	3.4% (2.8%, 4.2%)	0.82 (0.73, 0.92)		<0.001§
**Dynamic Education**	3.4% (2.6%, 4.5%)	2.6% (2.0%, 3.3%)	0.73 (0.60, 0.88)		
**Static Education**	5.0% (3.8%, 6.4%)	4.4% (3.4%, 5.7%)	0.89 (0.76, 1.04)	1.22 (0.93, 1.60)	0.151‡

§The p-value compares whether the overall change in the odds of reason for non-administration differs between pre-education and post-education, regardless of arm assignment.

‡The p-value compares whether the change in the odds of missing an administration differs by arm (i.e. a test of interaction between period and arm).

% = percent; CI = confidence interval.

### Nurse perceptions survey

**Fig 2:**
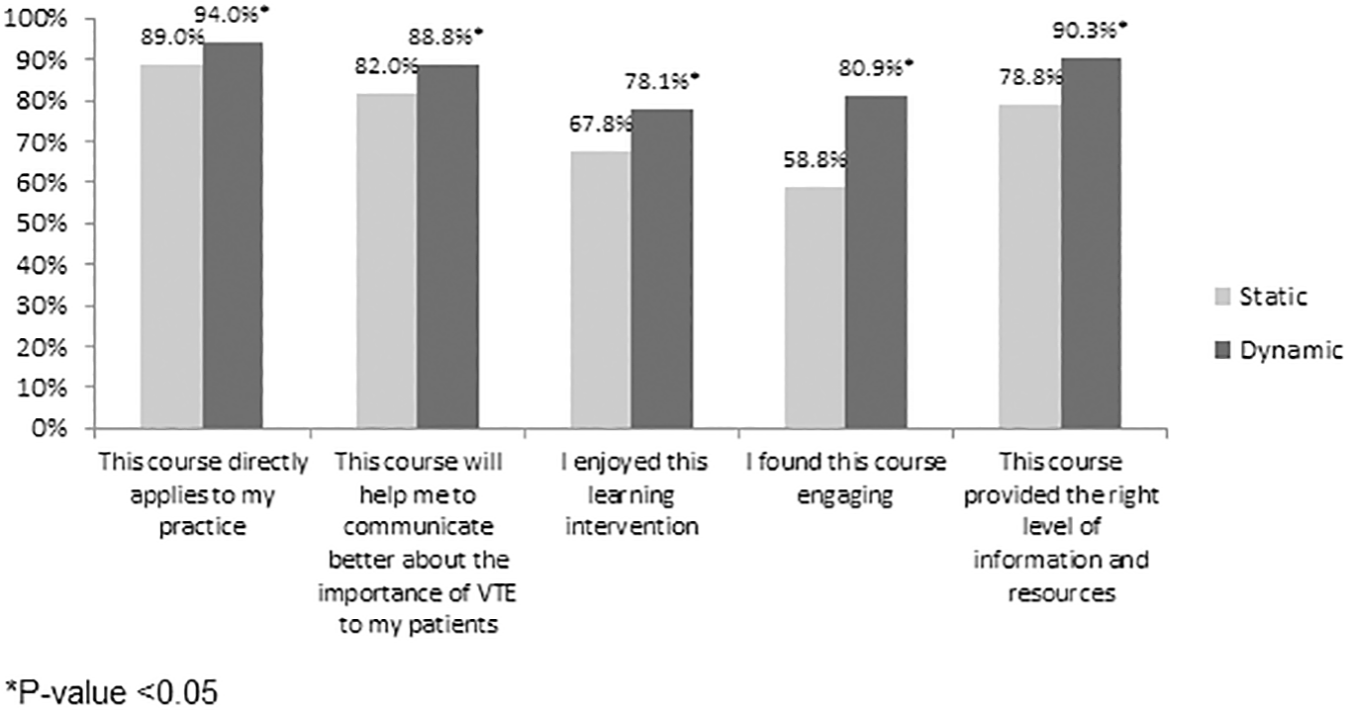
Nurse-reported satisfaction with and perception of Dynamic education module and the Static education module across five domains of engagement among nurses who completed their assigned education module.

Overall 580/801 (72.4%) of nurses, including 245/396 (61.9%) who completed the Static module and 335/405 (82.7%) who completed the Dynamic module responded to the voluntary follow-up survey. Scores were significantly higher in the Dynamic arm for all questions asked. Compared with nurses who completed the Static module, significantly more nurses reported that the Dynamic module was engaging (80.9% vs. 58.8%, p = 0.005), was enjoyable (78.1% vs. 67.8%, p = 0.005), helped to better communicate the importance of VTE to patients (88.8% vs. 82.0%, p = 0.020), provided the right level of information and resources (90.3% vs. 78.8%, p<0.001), and directly applied to their clinical practice (94.0% vs. 89.0%, p = 0.028, Fig 2).

## Discussion

Overall nurse education reduced the frequency of VTE prophylaxis non-administration. This effect was driven by reductions in non-administration due to causes other than patient or family request. Nurses found the interactive Dynamic module to be more engaging, enjoyable, and enable better patient engagement. While we were unable to demonstrate a statistically significant greater reduction in non-administration, the magnitude of improvement was slightly greater for the Dynamic module. These findings indicate that interactive, learner-centric education might be most appropriate to change practice and should be futher applied to other domains of clinical education.

This is the first study to rigorously study the comparative effectiveness of different nurse education approaches on clinical practices. A systematic review of interventions to improve VTE prevention practices reported that education strategies alone are not sufficient to drive and sustain change.[23] However, education is a necessary component of any successful intervention and our findings help to identify a strategy to effectively engage nurses in clinically relevant educational content on specific topics for delivery of high-quality care. Interventions to improve prescription of appropriate VTE prophylaxis in hospitals have been wildly successful at doing just that[24–28] and are promoted by AHRQ as one of the top ten most important patient safety practices.[5–8] However, improving prescription is only one step in a multi-step process to ensure defect-free VTE prevention; medication doses prescribed must be administered.[18]

This is not the first study to attempt to improve administration of prescribed VTE prophylaxis among hospitalized patients. One study at a large academic medical center relied on a clinical pharmacist engaging all patients prescribed pharmacologic VTE prophylaxis in an individualized education session. The investigators found that non-administration improved from 10.1% to 5.6%, though this intervention required one hour of a pharmacist’s time per patient, which is not sustainable.[12] Another study implemented a three-step approach to improve VTE prophylaxis administration that included a standardized nurses’ response to patient refusal of VTE prophylaxis, integration of daily assessment of VTE prophylaxis into a multidisciplinary rounds checklist, and frequent audit and feedback of unit performance. In this study, non-administration of prescribed VTE prophylaxis decreased from 24.7% to 14.7%.[13] While our study showed a more modest change in VTE prophylaxis medication non-administration, the burden of implementation and completion was also markedly lower. While education is an essential component for quality improvement interventions, education alone is not enough to elcit and sustain change. Future quality improvement efforts should include engaging education combined with more intensive, active interventions. Studies focused on giving individual physicians feedback about their prescribing habits have shown marked improvement in practice.[29–32] It is reasonable to believe that providing nurses with feedback about their administration practices would have a similar improvement on practice habits. This is the first study to specifically identify individual nurse data for medication administration, the first step towards being able to provide individualized feedback to nurses. Additionally, because no signficiant difference in patient or family member refusal was observed, it is possible that more intensive education should be provided to patients to ensure that they are empowered to make informed decisions about their care.[33]

This study had several strengths. We chose a clearly defined clinically-relevant outcome (i.e. dose non-administration) that was directly attributable to individual nurses before and after completion of the assigned education module. The education modules were relatively inexpensive and easily implemented using our hospital-wide learning management system. The interventions were effectively deployed and all relevant data were captured, including a high survey response rate from the nursing staff in both arms. Nurses, outcome assessors, and biostatisticians were blinded to minimize bias. In the Dynamic arm there was a particularly high response rate to the voluntary follow-up survey, suggesting that nurses desire more interactive and engaging education than what is provided by traditional linear static education using PowerPoint slides with voiceover.

There were some limitations to this study. First, although randomized, baseline performance was better among nurses in the Dynamic arm of the study compared with nurses in the Static arm. Despite this difference at baseline, a slightly greater magnitude of effect was observed among nurses who were assigned to the Dynamic arm although it was not statistically significant. Second, this trial was conducted in a single center potentially limiting the generalizability of our findings. Nonetheless, this is the first randomized study of which we are aware that compares the effectiveness of differing educational approaches for bedside nurses and illustrates important information about how nurses respond to education. Third, we did not explore differences associated with education by specific clinical department (e.g. medical vs. surgical nurses) in the current study as it was too small to detect such high order interactions. It is possible that differences in response to education between medical and surgical nurses may be due to the difference in perceived importance of VTE prophylaxis between medical and surgical nurses;[16] however, we stratified floors by clinical department for randomization to ensure similar representation. This would be worth consideration as part of future dissemination.

Education for nurses significantly improves clinical practice. Our findings suggest that education should be tailored to the learner rather than applied in a standardized manner. Dynamic learner-centered education may be more effective at engaging nurses and, when considering the corpus of evidence including a slightly greater magnitude of reduction in non-administration, may have the potential to be more effective for improving clinical practice. The improvement in administration practice was driven by reductions in non-administration due to causes other than patient or family refusal. Future studies will more accurately demonstrate improved effectiveness of this educational strategy, and should explore differences in the effect of education on different populations of learners, the sustainability of educational strategies on practice, and refine the education approach to optimize practice improvement.

## Supporting information

S1 File Protocol. (DOCX)

S2 File CONSORT checklist. (PDF)

S3 File Stata.do file for outputation analyses. (DO)

S4 File Stata.log file for outputation analyses. (LOG)
