# Supplementary material for: Effectiveness of two distinct web-based education tools for bedside nurses on medication administration practice for venous thromboembolism prevention: A randomized clinical trial
Source: PLoS One. 2017 Aug 16;12(8):e0181664. doi: 10.1371/journal.pone.0181664 (PMC5558918; doi:10.1371/journal.pone.0181664)
Supplement: S1 File — (DOCX) [file pone.0181664.s001.docx]

**Nurse Education Study Protocol**

**Title:** Educating Nurses About Venous Thromboembolism (VTE) Prevention

**Registration**: ClinicalTrial.gov NCT02301793

**Version**: 1 November 16, 2015

**Funding:** Patient Centered Outcomes Institute (PCORI)

The funders will have no role in the conduct of this study

**Contact Information**

Elliott R. Haut, MD, PhD, FACS

Associate Professor of Surgery, Anesthesiology / Critical Care Medicine (ACCM), Emergency Medicine, & Health Policy and Management

Division of Acute Care Surgery, Department of Surgery

The Johns Hopkins University School of Medicine

Sheikh Zayed 6107C, 1800 Orleans St.

Baltimore, MD 21287

410 502 3122 (phone)

410 502 3569 (fax)

ehaut1@jhmi.edu

**1. Introduction**

**Public Health Impact of Venous Thromboembolism**

Venous thromboembolism (VTE), comprised of deep vein thrombosis (DVT) and/or pulmonary embolism (PE), affects 350,000-600,000 individuals in the United States annually. More than 100,000 people die each year in the United States as a result of PE.^1^ Numerous studies have shown that VTE prophylaxis is vastly underutilized in hospitals^3, 4^ and the Agency for Healthcare Research and Quality (AHRQ) has listed strategies to improve VTE prevention on its top ten list for patient safety practices.^5-8^ Consequently, numerous interventions have been implemented to improve prescription of VTE prophylaxis^8-11^ with the implicit assumption that medications prescribed for hospitalized patients will always be administered.

**Deficits in Knowledge about Venous Thromboembolism**

In an attempt to improve venous thromboembolism (VTE) prophylaxis adherence we carried out qualitative studies to obtain patients' viewpoints on how nurses should be educated about VTE prevention and to assess nurses' beliefs and perceptions about pharmacologic VTE prophylaxis. We observed deficiencies in nurses' knowledge and misconceptions about VTE prophylaxis that likely lowers adherence to administration of prescribed VTE prophylaxis doses.

**Rationale for Education Trial**

As a part of our original Patient-Centered Outcomes Research Institute (PCORI) proposal, we planned to educate nurses to address the observed deficiencies and misconceptions and improve their ability to communicate effectively with patients. Historically, nurse education has been done via a linear, PowerPoint-based platform with voice-over but with no interactive component (Static). A newer platform for nurse education became available for use and includes scenario-based teaching, ongoing assessment, and immediate remediation. Most importantly, it is a highly interactive product (Dynamic).

**Study Objectives and Hypotheses**

The Objective of this study is to improve knowledge about the benefits and importance of VTE prophylaxis and harms associated with VTE with the goal of decreasing non-administration of prescribed VTE prophylaxis.

- Primary Hypothesis: Nurse participants who receive either of these interventions will improve administration of prescribed VTE prophylaxis evidenced by a decrease in frequency of non-administered doses of VTE prophylaxis, compared with their frequency at baseline.
- Secondary Hypothesis: Nurse participants who receive the Dynamic education format will have a larger decrease in frequency of missed doses of VTE prophylaxis compared with those who participate in the Static education format.
  1. **Study Design**

1. Study Design
   1. Cluster randomized trial
2. Study setting: Johns Hopkins Hospital, Baltimore MD
3. Eligibility Criteria
   1. All nurses permanently assigned to one of the 21 included study floors
4. Interventions
   1. Education A: An interactive dynamic learner-centric scenario-based education module with ongoing assessment, and immediate remediation (Dynamic).
   2. Education B: A linear static PowerPoint-based platform with voice-over but with no interactive component (Static).

Participants will be assigned one of two education modules based on their permanent floor assignment. The same education type will be assigned to all nurses on a single floor to mitigate issues related to contamination if nurses discuss the education with their colleagues.

1. Enrollment
   1. Nurses will be identified using our centralized education directory that associates nurses with their designated departments and hospital floors. All nurses permanently assigned to one of the 21 included study floors will be included in this study. Nurses who are not permanently associated with one of the 21 hospital floors (e.g. traveling nurse, float nurse) will be excluded from this study.
2. Randomization
   1. Nurses will be identified using our centralized education directory that associates nurses with their designated departments and hospital floors. Nurses will be cluster randomized by floor to receive one of two education modules about VTE prevention. Because of known differences between medical and surgical floors in VTE prophylaxis administration practice and culture, floors were stratified by department (i.e. medicine and surgery) for randomization. Within strata, we will use a coin toss to randomize floors into study arms.
3. Duration of Study
   1. Nurses will be given three months to complete the assigned education module. Data will be collected for one full year to include medication administration practice at baseline, during the education trial, and after the education trial.
4. Outcomes
   1. Primary Outcome measure: Proportion of non-administered doses of pharmacological VTE prophylaxis (dose level)
   2. Secondary Outcome Measures:
      1. Nurse-reported relevance of and satisfaction with the education modules
      2. Proportion of doses documented as missed due to patient refusal (dose level)
      3. Proportion of patients with any VTE (patient level)
         1. VTE are defined by AHRQ PSI-12 diagnosis codes
      4. Proportion of patients with Deep Vein Thrombosis (DVT) (patient level)
      5. Proportion of patients with Pulmonary Embolism (PE) (patient level)

Patient demographic data will be extracted from the Johns Hopkins Hospital administrative database. Pharmacologic VTE prophylaxis medication administration data will be extracted directly from electronic medication administration record in our computerized provider order entry system. Immediately following completion of the assigned education module, nurses will be asked to complete a voluntary 5-question survey to assess the relevance of and satisfaction with the education module.

- 1. **Analytic methods**

1. Baseline Characteristics
   1. Comparison of both arms to ensure similarity at baseline
   2. Descriptive analysis of baseline characteristics i.e. simple counts and proportions by trial arm
2. Multi-level mixed effects linear regression

Due to the complexity of the multilevel structure of the data (i.e. multiple doses per patient across various hospitalizations, nurses and floors), multiple outputation^19^ will be used to reduce the levels of hierarchical structure to the floor level and nurse level by randomly selecting one dosage per patient. By reiterating the procedure 1000 times, we will estimate the odds ratios (ORs) and 95% confidence intervals conditional on the floor and nurse.

- 1. Primary analyses: Intent-to-Treat
  2. Secondary analyses
     1. Per protocol: Nurses who complete the education where the date of completion serves as an individual inflection point for pre-post education
     2. Nurse-reported relevance of and satisfaction with the education modules will be analyzed using a two-sided Chi-squared test

**References**

1. US Department of Health and Human Services. The Surgeon General’s Call to Action to Prevent Deep Vein Thrombosis and Pulmonary Embolism. <http://www.ncbi.nlm.nih.gov/books/NBK44178/>. Accessed April 5, 2014.

2. Goldhaber SZ, Tapson VF, DVT FREE Steering Committee. A prospective registry of 5,451 patients with ultrasound-confirmed deep vein thrombosis. *The American Journal of Cardiology*. 2004;93(2):259-262.

3. Cohen AT, Tapson VF, Bergmann J, et al. Venous thromboembolism risk and prophylaxis in the acute hospital care setting (ENDORSE study): a multinational cross-sectional study*. The Lancet*. 2008;371(9610):387-394. doi: 10.1016/s0140-6736(08)60202-0.

4. Shekelle PG, Wachter RM, Pronovost PJ, Schoelles K, McDonald KM, Dy SM, Shojania K, Reston J, Berger Z, Johnsen B, Larkin JW, Lucas S, Martinez K, Motala A, Newberry SJ, Noble M, Pfoh E, Ranji SR, Rennke S, Schmidt E, Shanman R, Sullivan N, Sun F, Tipton K, Treadwell JR, Tsou A, Vaiana ME, Weaver SJ, Wilson R, Winters BD. Making Health Care Safer II: An Updated Critical Analysis of the Evidence for Patient Safety Practices. 2013;211.

5. Shekelle PG, Pronovost PJ, Wachter RM, et al. The top patient safety strategies that can be encouraged for adoption now*. Ann Intern Med*. 2013;158(5 Pt 2):365-368. doi: 10.7326/0003-4819-158-5-201303051-00001; 10.7326/0003-4819-158-5-201303051-00001.

6. Haut ER, Lau BD. Prevention of Venous Thromboembolism: Brief Update Review. In: *In "Making Health Care Safer II: An Updated Critical Analysis of the Evidence for Patient Safety Practices.* Rockville, MD.: Agency for Healthcare Research and Quality; 2013.

7. Lau BD, Haut ER. Practices to prevent venous thromboembolism: A brief review*. BMJ Qual Saf*. Mar 2014;23(3):187-195.

8. Durieux P, Nizard R, Ravaud P, Mounier N, Lepage E. A Clinical Decision Support System for Prevention of Venous Thromboembolism Effect on Physician Behavior*. JAMA*. 2000;283(21):2816-21.

9. Streiff MB, Carolan H, Hobson DB, et al. Lessons from the Johns Hopkins Multi-Disciplinary Venous Thromboembolism (VTE) Prevention Collaborative*. BMJ*. 2012;344:e3935.

10. Kucher N, Koo S, Quiroz R, et al. Electronic alerts to prevent venous thromboembolism among hospitalized patients. *N Engl J Med*. 2005;352(10):969-77.

11. Follmann D, Proschan M, Leifer E. Multiple outputation: inference for complex clustered data by averaging analyses from independent data*. Biometrics*. 2003;59(2):420-429.
